# A Systematic Review of the Current Evidence of the Effectiveness and Safety of Immunoprophylaxis Using Sublingual Dead Whole Bacteria to Prevent Recurrent Urinary Tract Infections (rUTIs)

**DOI:** 10.3390/antibiotics15010006

**Published:** 2025-12-19

**Authors:** José Medina-Polo, Ana Arrébola-Pajares, Eva Falkensammer, Zafer Tandogdu

**Affiliations:** 1 Department of Urology, Hospital Universitario 12 de Octubre Imas12 & Universidad Complutense de Madrid, 28040 Madrid, Spain; 2 Department of Urology, Klinikum Wels-Grieskirchen, 4710 Wels, Austria; eva.falkensammer@gmail.com; 3 Department of Urology, University College London Hospitals, London NW1 2BU, UK; 4 Division of Surgery and Interventional Science, University College London, London WC1E 6BT, UK

**Keywords:** antibiotics, immunoprophylaxis, meta-analysis, recurrent urinary tract infection, systematic review

## Abstract

**Background/Objectives**: Recurrent urinary tract infections (rUTIs) remain a major clinical challenge, but sublingual immunoprophylaxis with inactivated whole bacteria is a promising alternative to antibiotic prophylaxis. The objective of this systematic review was to assess the efficacy and safety of sublingual bacterial vaccines. **Methods**: We searched MEDLINE, Cochrane CENTRAL, and Embase (January 1979–August 2025) for English-language studies evaluating bacterial vaccines for rUTI prevention. Eligible studies included adults receiving sublingual immunoprophylaxis with heat-inactivated whole bacteria. Outcomes were UTI recurrence, infection-free interval, and adverse events. Both MV140 and autovaccines were assessed. **Results**: Of the 262 records identified, 14 studies met the inclusion criteria (4 comparatives, including 1 randomized trial, and 10 observational studies). UTI incidence decreased from 3.2–6.8 to 0–1.5 episodes/year at 12 months. The proportion of UTI-free patients ranged from 9.8 to 90% with immunoprophylaxis versus 0 to 25% with antibiotics or placebo. At 12 months, UTI-free rates were 10–49% with autovaccines and 9.7–60% with MV140. Patients with ≥3 UTIs ranged from 14.3 to 60.2% and 20 to 56.1% with autovaccines and MV140, respectively. The RCT reported adverse events in 0–40.8% of treated patients and 50% of placebo patients, predominantly mild. **Conclusions**: Although the available evidence is heterogeneous and largely derived from observational studies, sublingual immunoprophylaxis with heat-inactivated whole-bacteria—either standardized (MV140) or tailored to urine culture results—appears to be an effective and safe strategy for reducing the frequency of rUTI and prolonging infection-free intervals. However, larger randomized trials are required to confirm these findings.

## 1. Introduction

Recurrent urinary tract infections (rUTIs) are defined as at least three episodes of cystitis per year or two episodes of cystitis in six months [1]. It is estimated that up to 3% of women experience rUTIs [1]. rUTIs negatively affect patients’ quality of life, leading to a reduction in social and sexual relationships, diminished self-esteem, and impaired work capacity [2]. Prevention of rUTIs includes counseling on risk factor avoidance, non-antimicrobial strategies, and antimicrobial prophylaxis. Non-antibiotic preventive measures are recommended over antibiotic therapy to avoid antimicrobial resistance and other adverse effects associated with antibiotic use. Immunoprophylaxis using whole bacteria or bacterial components is an alternative strategy for preventing rUTIs [1]. Several immunoprophylactic approaches have been developed to stimulate the immune system via dendritic cells and T-cell-mediated cytokine production [3,4,5,6]. Formulations include oral, sublingual, intramuscular, and intravaginal preparations (StroVac, OM-89, ExPEC4V, MV140, and Solco-Urovac). Previous systematic reviews have evaluated these different agents [7,8]. The composition and mechanism of action of individual immunomodulatory agents are sufficiently different such that pooled analyses of results from these disparate trials are unlikely to provide robust conclusions. Therefore, each preparation should be reviewed separately [8]. Recently, interest in sublingual inactivated whole bacteria for rUTI prevention has increased, particularly with the MV140 formulation. MV140 (Uromune, Q Pharma/Inmunotek S.L.) is a glycerinated suspension of whole-cell, heat-inactivated bacteria [8,9]. The original formula consists of equal amounts of four common UTI-causing microorganisms: *Escherichia coli*, *Klebsiella pneumoniae*, *Proteus vulgaris,* and *Enterococcus faecalis*. Since 2017, the only approved formulation available in Spain has been the autovaccine. Autovaccination consists of heat-inactivated whole bacteria tailored to a patient’s urine culture results. Both immunoprophylactic preparations are administered sublingually as two daily puffs of 100 μL each (108 bacteria/puff), with no concomitant food or beverage intake [10].

The aim of this systematic review and meta-analysis was to evaluate the current evidence on the effectiveness and safety of immunoprophylaxis using sublingual inactivated whole bacteria (MV140 and autovaccination) to prevent rUTIs. The main outcomes were UTI incidence at 12 months and the proportion of patients who remained UTI-free.

## 2. Results

**Figure 1:**
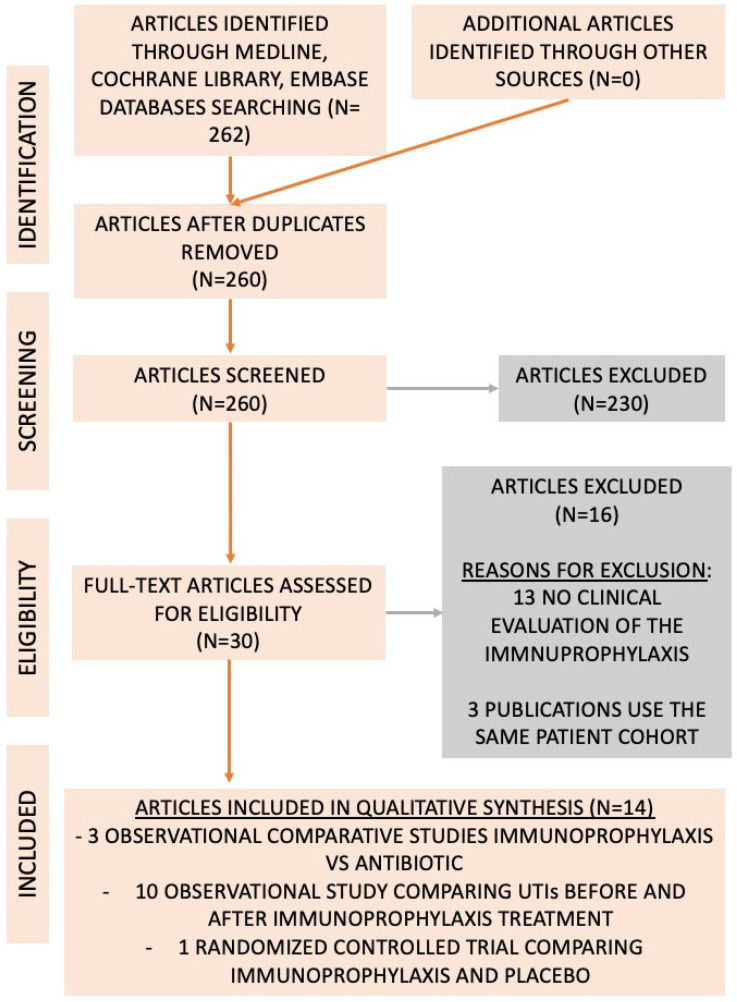
Flowchart with the number of publications evaluated and included in the analysis, according to the PRISMA guidelines.

The literature search identified 262 studies evaluating the use of sublingual immunoprophylaxis with inactivated whole bacteria to manage rUTIs. Of these, 232 were excluded because they did not assess sublingual immunoprophylaxis with heat-inactivated whole bacteria. A total of 30 full-text articles were reviewed for eligibility, and 14 studies were included (4 comparative and 10 observational studies) [10,11,12,13,14,15,16,17,18,19,20,21,22,23]. Details of the literature search and reasons for exclusion are summarized in Figure 1.

### 2.1. Comparative Studies

Four comparative studies were included. Three were observational studies using antibiotic prophylaxis as the control [11,12,18], and one was an RCT comparing the MV140 formulation with placebo [16]. The number of patients in the treatment arms ranged from 139 to 360. Study characteristics are summarized in Table 1. Across these studies, the mean annual incidence of UTIs decreased from 3.2–6 episodes at baseline to 0–1.35 episodes at 12 months in patients receiving immunoprophylaxis [11,12,16]. The proportion of patients who remained UTI-free at 12 months was 56–90% in the immunoprophylaxis group, 0–2.5% in those managed with low-dose antibiotics, and 25% in those managed with a placebo. The RCT reported adverse events in 40.8% of patients receiving immunoprophylaxis and 50% receiving placebo (205 adverse reactions in 101 patients) [11,12,16,18]. The most common adverse events were chest infections (2.6%), candidiasis (2.6%), and vaginitis (2.0%). A total of 5 of 152 patients (3.2%) reported serious adverse events unrelated to immunoprophylaxis [16]. A total of 9 of the 101 adverse events were considered adverse reactions related to the study intervention (2 in the placebo group and 3 in MV140). The number of patients who withdrew from the study due to adverse events was 2 (2.5%) in the placebo group and 5 (6.2%) in the MV140-treated group [24]. Several studies also reported reduced isolation of non-*Escherichia coli* pathogens, lower antimicrobial resistance rates, and improved quality of life with immunoprophylaxis [11,12,16]. In the study by Lorenzo et al., *Klebsiella* spp. isolation was 12% in the antibiotic prophylaxis group and 0% in the immunoprophylaxis group. Furthermore, the percentage of bacteria resistant to different antibiotics was 31% and 45% in those receiving immunoprophylaxis and antibiotic prophylaxis, respectively [12]. The main findings from comparative studies are summarized in Table 2.

### 2.2. Observational Studies

Ten observational studies were included, all comparing UTI incidence before and after immunoprophylaxis [10,13,14,15,17,19,20,21,22,23]. Although both men and women were studied, women accounted for more than 65% of cases overall. In the study by Bonillo-García et al., which focused on patients with neurogenic bladder, men comprised 68% of the cohort [23]. Treatments included both the MV140 formulation (1228 cases) and autovaccination (1367 cases). Study characteristics are summarized in Table 3. The mean annual UTI incidence decreased from 3.7–6.8 episodes at baseline to 0.2–1.58 episodes after 12 months of immunoprophylaxis [14,15,17,19,20,22]. Reported UTI reduction at 12 months ranged from 9.8% to 78%, and the proportion of patients experiencing ≥3 UTIs at follow-up ranged from 14.3% to 57.6% [10,13,14,17,19,20,22,23]. Adverse events occurred in 0–14% of patients and were generally mild. Adverse effects include skin rash, mucosal burning, upper gastrointestinal discomfort, and fatigue [10,13,14,15,17,19,20,22]. Several studies also reported reduced healthcare costs and improved patient quality of life with immunoprophylaxis [17,20,21]. The main findings from observational studies are summarized in Table 4.

Autovaccines were evaluated explicitly in six studies (1493 patients) [10,14,18,21,22,23]. Characteristics are summarized in Table 5. The mean annual incidence of UTIs decreased from 3.7–5 episodes at baseline to 0.3–0.98 episodes at 12 months [14,22]. At 12-month follow-up, UTI-free rates ranged from 10 to 49% for autovaccines and 9.7 to 60% for MV140, while the proportion of patients with ≥3 UTIs ranged from 14.3 to 60.2% for autovaccines and 20 to 56.1% for MV140 [10,14,22]. Reported adverse events with autovaccination were rare (0–1.3%) and mild [10,15,18,22]. In patients with neurogenic bladders, Bonillo-García et al. reported improved quality of life following autologous vaccine therapy [23]. The main findings from autovaccine studies are summarized in Table 6.

### 2.3. Metanalysis

**Figure 2:**
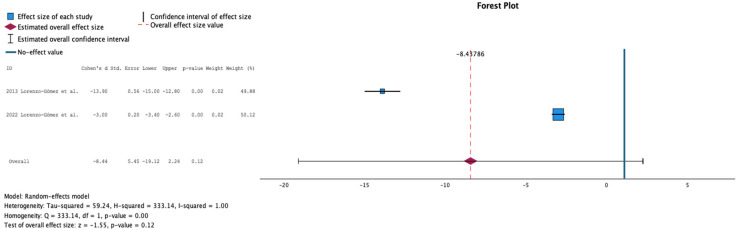
Incidence of Urinary Tract Infections at 12 Months Follow-Up in Comparative Studies [11,16].

Figure 2 shows the meta-analysis comparing placebo or antibiotic prophylaxis with sublingual immunoprophylaxis using the MV140 formulation for the outcome of UTI incidence. Two studies were included, comprising 160 patients in the antibiotic group, 76 in the placebo group, and 298 in the immunoprophylaxis group. No significant reduction in UTI incidence was observed with immunoprophylaxis compared with controls (mean difference: −8.44; 95% CI: −19.12 to 2.24; p = 0.12). Statistical heterogeneity was high (Q = 333.14, *p* < 0.001; τ^2^ = 52.24; H^2^ = 333.14; I^2^ = 99%), indicating considerable variability between studies.

**Figure 3:**
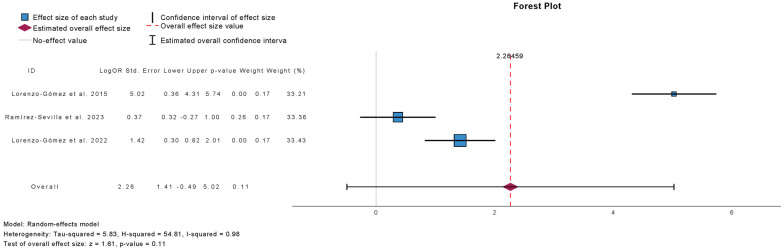
Incidence of No Urinary Tract Infections at 6 Months Follow-Up in Comparative Studies [12,16,18].

Figure 3 presents the meta-analysis comparing placebo or antibiotic prophylaxis with sublingual immunoprophylaxis using the MV140 formulation for the outcome of UTI-free prevalence at 6 months. Three studies were included, comprising 465 patients in the antibiotic group, 76 in the placebo group, and 750 in the immunoprophylaxis group. Immunoprophylaxis did not significantly increase the proportion of UTI-free patients at 6 months compared with controls (mean difference: 2.27; 95% CI: −0.49 to 5.02; *p* = 0.11). Heterogeneity was substantial (τ^2^ = 5.83; H^2^ = 54.81; I^2^ = 98%), reflecting considerable variability across studies.

**Figure 4:**
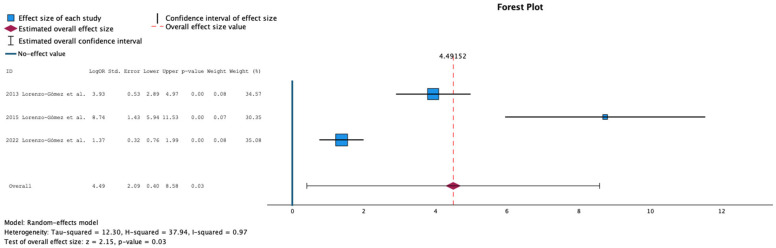
Incidence of No Urinary Tract Infections at 12 Months Follow-Up in Comparative Studies [11,12,16].

Figure 4 summarizes the meta-analysis comparing placebo or antibiotic prophylaxis with sublingual immunoprophylaxis using the MV140 formulation for the outcome of UTI-free prevalence at 12-month follow-up. Three studies were included, comprising 499 patients in the antibiotic group, 76 in the placebo group, and 558 in the immunoprophylaxis group. Immunoprophylaxis was associated with a significantly higher proportion of UTI-free patients at 12 months compared with controls (mean difference: 4.49; 95% CI: 0.40 to 8.58; *p* = 0.03). Heterogeneity was substantial (τ^2^ = 12.30; H^2^ = 34.94; I^2^ = 97%), indicating considerable variability across studies.

**Figure 5:**
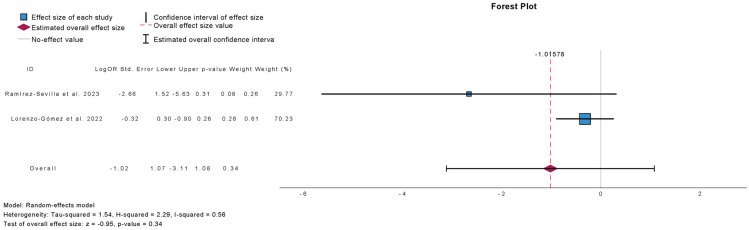
Incidence of Side Effects in Comparative Studies [16,18].

Figure 5 presents the meta-analysis comparing placebo or antibiotic prophylaxis with sublingual immunoprophylaxis using the MV140 formulation for the outcome of side-effect prevalence. Two studies were included, comprising 126 patients in the antibiotic group, 76 in the placebo group, and 390 in the immunoprophylaxis group. There was no significant difference in the prevalence of side effects between immunoprophylaxis and control groups (mean difference: −1.02; 95% CI: −3.11 to 1.08; *p* = 0.34). Heterogeneity was low (τ^2^ = 1.54; H^2^ = 2.29; I^2^ = 56%), indicating modest variability across studies. 

**Figure 6:**
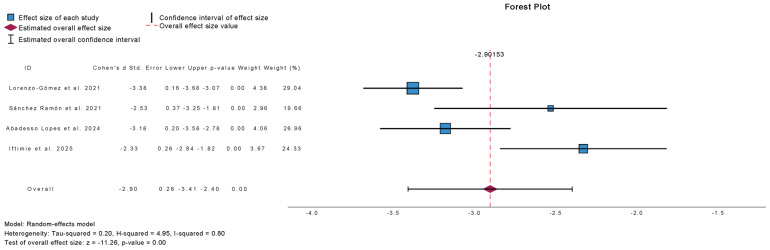
Incidence of Urinary Tract Infections at 12 Months Follow-Up in Observational Studies (Pre- and Post-Treatment) [14,15,19,22].

Figure 6 summarizes the meta-analysis of UTI incidence at 12-month follow-up in studies comparing outcomes before and after treatment with sublingual immunoprophylaxis. Four studies were included, comprising 384 patients. Immunoprophylaxis was associated with a significant reduction in UTI incidence at 12 months (mean difference: −2.91; 95% CI: −3.41 to −2.40; *p* < 0.01). Heterogeneity was substantial (τ^2^ = 0.20; H^2^ = 4.95; I^2^ = 80%), reflecting moderate variability across studies.

**Figure 7:**
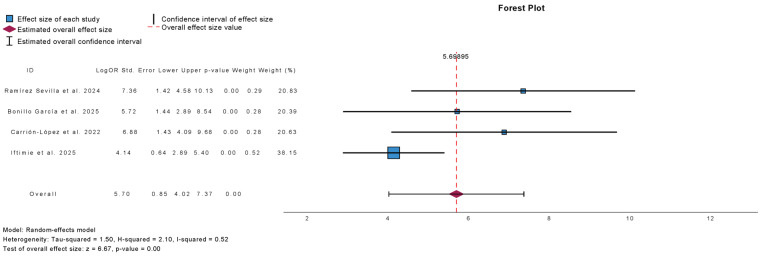
Incidence of No Urinary Tract Infections at 3 Months Follow-Up in Observational Studies (Pre- and Post-Treatment) [10,17,22,23].

Figure 7 presents the meta-analysis of UTI-free prevalence at 3-month follow-up in studies comparing outcomes before and after treatment with sublingual immunoprophylaxis. Four studies were included, comprising 1389 patients. Immunoprophylaxis was associated with a significant increase in the proportion of UTI-free patients at 3 months (mean difference: 5.70; 95% CI: 4.02 to 7.37; *p* < 0.01). Heterogeneity was moderate (τ^2^ = 1.50; H^2^ = 2.10; I^2^ = 52%), indicating some variability across studies. 

**Figure 8:**
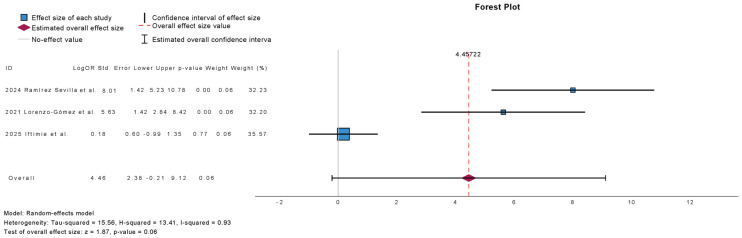
Incidence of More than 3 Urinary Tract Infections at 12 Months Follow-Up in Observational Studies (Pre- and Post-Treatment) [10,14,22].

Figure 8 summarizes the meta-analysis of the prevalence of patients experiencing more than three UTIs at 12-month follow-up in studies comparing outcomes before and after treatment with sublingual immunoprophylaxis. Three studies were included, comprising 1353 patients. There was no statistically significant difference in the prevalence of patients with more than three UTIs at 12 months (mean difference: 4.46; 95% CI: −0.21 to 9.12; *p* = 0.06). Heterogeneity was high (τ^2^ = 15.56; H^2^ = 13.41; I^2^ = 93%), indicating considerable variability across studies.

**Figure 9:**
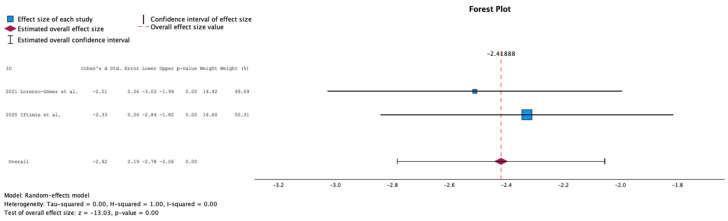
Incidence of Urinary Tract Infections at 12 Months Follow-Up in Studies Evaluating Autovaccine [14,22].

Figure 9 summarizes the meta-analysis of UTI incidence at 12-month follow-up in studies evaluating autovaccine. Two studies were included, comprising 109 patients. Autovaccine treatment was associated with a significant reduction in UTI incidence at 12 months (mean difference: −2.42; 95% CI: −2.78 to −2.06; *p* < 0.01). 

**Figure 10:**
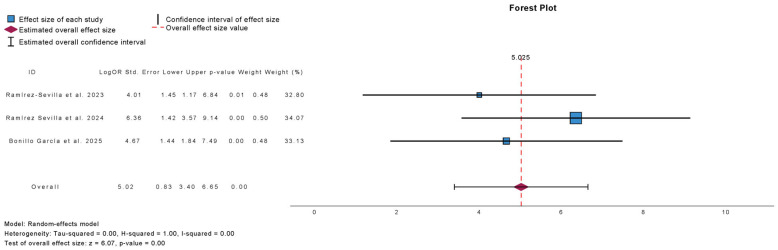
Incidence of No Urinary Tract Infections at 6 Months Follow-Up in Observational Studies Evaluating Autovaccine [10,18,23].

Figure 10 summarizes the meta-analysis of UTI-free prevalence at 6-month follow-up in studies evaluating autovaccine. Three studies were included, comprising 652 patients. Autovaccine treatment was associated with a significant increase in the proportion of UTI-free patients at 6 months (mean difference: 5.03; 95% CI: 3.40 to 6.65; *p* < 0.01). 

**Figure 11:**
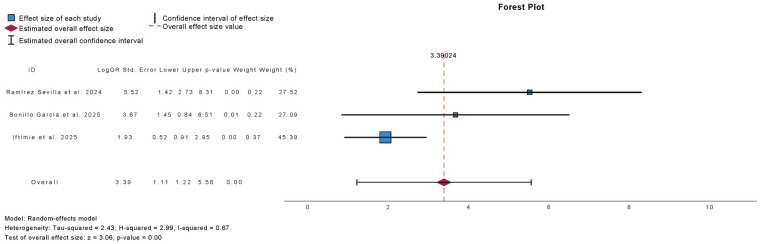
Incidence of No Urinary Tract Infections at 12 Months Follow-Up in Observational Studies Evaluating Autovaccine [10,22,23].

Figure 11 summarizes the meta-analysis of UTI-free prevalence at 12-month follow-up in studies evaluating autovaccine. Three studies were included, comprising 575 patients. Autovaccine treatment was associated with a significant increase in the proportion of UTI-free patients at 12 months (mean difference: 3.39; 95% CI: 1.22 to 5.56; *p* < 0.01). Heterogeneity was moderate (τ^2^ = 2.43; H^2^ = 2.99; I^2^ = 67%). 

**Figure 12:**
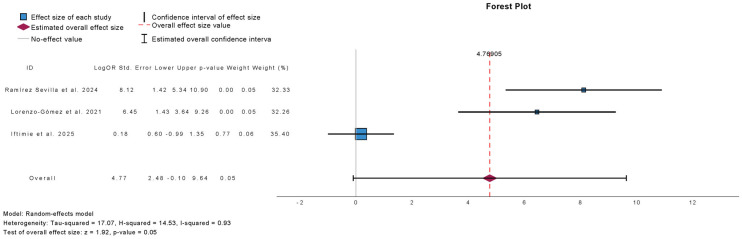
Incidence of More than 3 Urinary Tract Infections at 12 Months Follow-Up in Observational Studies Evaluating Autovaccine [10,14,22].

Figure 12 summarizes the meta-analysis of the prevalence of patients with more than three UTIs at 12-month follow-up in studies evaluating autovaccine. Three studies were included, comprising 565 patients. Autovaccine treatment was associated with a borderline significant reduction in the prevalence of patients with >3 UTIs at 12 months (mean difference: 4.82; 95% CI: −0.10 to 9.64; *p* = 0.05). Heterogeneity was high (τ^2^ = 17.07; H^2^ = 14.53; I^2^ = 93%). 

### 2.4. Risk of Bias Assessment

**Figure 13:**
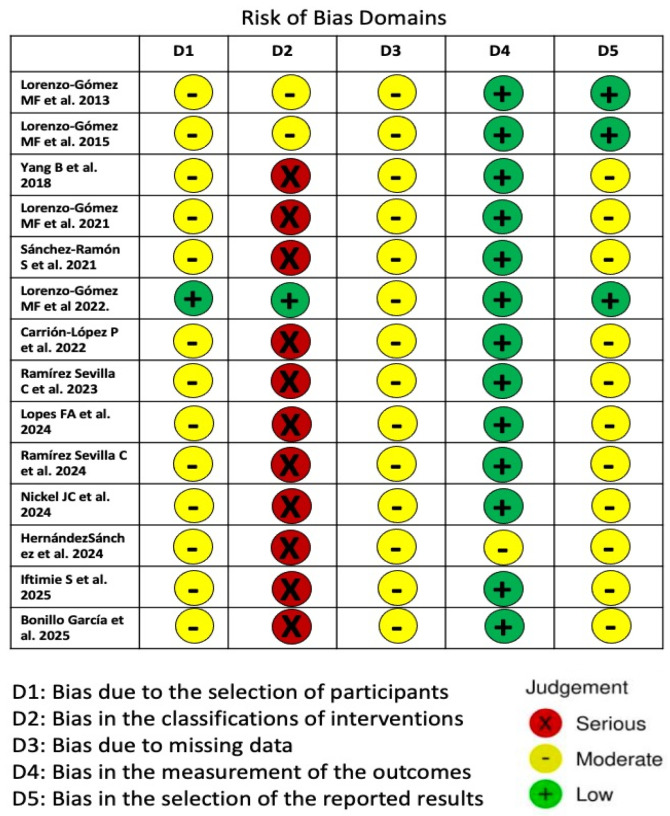
Risk of bias assessment for the nine included randomized comparative and observational studies utilizing the ROBINS-I tool [10,11,12,13,14,15,16,17,18,19,20,21,22,23].

Patient selection and classification represented potential sources of bias in the included studies. Most studies assessed the efficacy of immunoprophylaxis using a pre- and post-treatment comparison. In the comparative studies, the criteria for treatment allocation were often unclear, as decisions were based on physician judgment, which may have influenced outcomes [24,25]. Only the randomized controlled trial, including 240 participants, had a low risk of bias regarding patient classification [16]. All studies had a low to moderate risk of bias concerning selective outcome reporting. A summary of the risk of bias for the included studies is presented in Figure 13.

### 2.5. Evidence Synthesis

This review evaluates the efficacy and safety of immunoprophylaxis with sublingual, inactivated whole bacteria for the prevention of rUTIs, including fourteen studies with a total of 3504 patients receiving immunoprophylaxis. Two formulations were evaluated: MV140, which consists of *Escherichia coli*, *Klebsiella pneumoniae*, *Proteus vulgaris*, and *Enterococcus faecalis***,** and an autovaccine, prepared based on individual urine culture results. The autovaccine was evaluated in 1453 patients. Most studies assessed efficacy at 12 months of follow-up. Although UTIs frequently occurred in the follow-up, immunoprophylaxis was associated with a reduction in UTI incidence, ranging from 10% to 80% at 12 months. Ramírez-Sevilla et al. reported that patients with more than five UTIs per year had a higher incidence of infections and recommended extending immunoprophylaxis by an additional three months [8,18]. Comparative efficacy between MV140 and autovaccine has also been evaluated. Both formulations demonstrated efficacy from 3 to 12 months post-treatment. Published results suggest that MV140 may achieve higher proportions of UTI-free patients at 12-month follow-up (9.7–60% for MV140 vs. 10–49% for the autovaccine) [10,14,22]. Similarly, the proportion of patients experiencing more than three UTIs at 12 months ranged from 20% to 56% for MV140 and 14% to 90% for autovaccine [10,14,22]. Reported side effects were mostly mild and local, with the autovaccine associated with slightly fewer adverse events [10,18,26].

## 3. Discussion

The systematic review of the evidence indicates that prevention of rUTIs with sublingual formulations of inactivated whole bacteria or autovaccines appears to be a promising strategy with high efficacy, which could avoid low-dose prophylaxis. Non-antibiotic treatment should be considered first-line management for the prevention of rUTIs [1,27,28]. Two prior systematic reviews have assessed the effect of immunoprophylaxis in patients with rUTIs. However, the analysis was conducted using different types of preparations. Different administration routes, mechanisms of action, and dosages may influence clinical outcomes [16,29,30,31]. In the meta-analysis by Mak et al., 16 studies were evaluated, 4 of which used the MV140 formula. Similarly, in the meta-analysis by Pratley et al., 3 of 17 studies reported outcomes using MV140 [7,8]. The present systematic review included 14 studies involving 3504 patients. Moreover, many studies assessing the efficacy of immunoprophylaxis had short follow-up periods and were predominantly conducted using female populations [7].

The focus of this systematic review was to analyze the efficacy of sublingual dead whole-bacteria formulations in preventing rUTIs, including the MV140 formula (*Escherichia coli*, *Klebsiella pneumoniae*, *Proteus vulgaris*, and *Enterococcus faecalis*) and autovaccines. The proposed mechanism of action involves modulation of the innate and adaptive immune response via dendritic cells in the sublingual epithelium. Activation of these dendritic cells induces a T-lymphocyte-mediated response, producing Th1, Th17, and IL-10 cytokines. Sublingual administration avoids intestinal metabolism, and multiple studies in both animal models and humans have demonstrated effective immune activation [3,4,5,6,32].

Most evidence on the efficacy of sublingual immunoprophylaxis is derived from studies comparing UTI incidence before and after treatment. Across more than 3000 patients, reductions in UTI incidence were observed at 12-month follow-up [10,12,14,17,22,23,24]. Although the incidence of urinary tract infections (UTIs) has decreased, it should be noted that UTIs may still occur after treatment. Studies by Lorenzo-Gómez et al. reported that the proportion of patients remaining UTI-free at 12 months ranged from 56% to 90% in the immunoprophylaxis group, compared with 0–2.5% in the low-dose antibiotic group; these data are derived from observational studies [11,12,14]. The Cochrane review on the efficacy of low-dose prophylaxis for recurrent UTIs reported recurrence rates of 0 to 0.9 episodes per patient-year during antibiotic prophylaxis. After discontinuation of prophylaxis, recurrence rates based on microbiological criteria were 1.2 episodes per patient-year in patients receiving nitrofurantoin and 1.3 episodes per patient-year in those receiving cotrimoxazole [33]. Data from an RCT reported a 12-month follow-up prevalence of UTI-free patients of 56% for the MV140 formulation and 25% in the placebo group [16]. These findings are concordant with other studies evaluating alternative strategies for the management of rUTIs, such as the ALTAR study assessing the use of methenamine hippurate [34].

The design of most studies carries a risk of reporting bias, as they lacked a comparator group or were retrospective [9,10]. In some studies, treatment choice was left to the clinician’s discretion, introducing potential group heterogeneity. Studies comparing UTI rates before and after immunoprophylaxis may also be prone to bias owing to improvements attributable to factors such as spontaneous changes in risk factors over time and behavioral modifications (e.g., hydration and sexual activity). Publication bias may also be present, with a tendency for studies showing positive results to be published preferentially. However, although much of the research has been conducted by a limited number of authors, the data have been followed prospectively and results updated periodically in some series [10,15,18,26]. Finally, the definition of UTI used in these studies generally required the presence of symptoms and a positive urine culture using a threshold of >10^5^ CFU/mL. However, in some studies, the threshold was not specified. In patients with typical UTI symptoms, or in those with urinary catheters, a lower threshold of ≥10^3^ CFU/mL may also be acceptable [1].

Sublingual immunoprophylaxis was predominantly evaluated in women, with only 185 men included in the studies [14,18,19,22,23]. A substantial proportion of participants were postmenopausal women, 1738 out of 2601 (66.8%). Hormonal status influenced outcomes: premenopausal women had higher UTI-free rates (14/22, 63.6%) compared to postmenopausal women (13/42, 33.3%; *p* = 0.012) [20]. Conversely, Ramírez et al. reported that postmenopausal women experienced a greater reduction in UTI episodes than premenopausal women (74.7% vs. 59.4%, *p* = 0.029) [19]. Lorenzo-Gómez et al. reported efficacy in older patients with both MV140 and autovaccine formulations, with enhanced outcomes following booster doses. No significant sex differences were observed, although both formulations appeared more effective in females [14]. Sub-analyses indicated comparable efficacy among smokers and patients with metabolic syndrome, while risk factors for UTIs included urostomy, chronic kidney disease, and immunosuppression [22,35].

Two different immunoprophylaxis approaches using sublingual administration of whole inactivated bacteria were analyzed: the standardized MV140 formulation and tailored autovaccines derived from the patient’s urine culture. The individual analysis of both formulations was evaluated in the research conducted by Ramírez et al. [10,18]. Among patients receiving MV140, reported efficacy rates were 95.8%, 88.4%, and 56.1% at 3, 6, and 12 months of follow-up, respectively. In patients treated with autovaccines, efficacy rates were 85.7%, 73.6%, and 60.2% at the same follow-up intervals. Moreover, the same research group reported that patients presenting with five or more UTIs experienced lower efficacy (80.2%, 64.3%, and 40%) compared to those with fewer than five UTIs (97.7%, 91.1%, and 64.7%) at 3, 6, and 12 months of follow-up (n = 611 with 12-month follow-up) [10]. Therefore, patients must be informed about the risk of recurrence. The one-year recurrence rates were 79.2% and 94.9% in patients with fewer than five and more than five UTIs prior to treatment, respectively. The authors recommended that immunoprophylaxis be maintained for more than three months in patients with more than five UTIs per year [10].

Quality of life (QoL) outcomes improved significantly. At 12 months, 80.3% of patients reported moderate or marked improvement, 58.1% were satisfied or delighted, and mean QoL scores increased by 1.5 points (21). Bonillo-Garcia et al. reported that neurological patients indicated “Improved greatly” (30.4%) or “Improved” (43.5%), with only 2.9% reporting deterioration [23].

Economic analyses suggest that sublingual immunoprophylaxis may be more cost-effective than both antibiotic and non-antibiotic adjuvant measures at reducing emergency visits, follow-up appointments, infection recurrence, and diagnostic testing [17,21].

Most studies reported an adverse event rate of less than 5%, with the majority of events being mild and including skin rash, mucosal irritation, gastrointestinal discomfort, and fatigue [14,17,19,20]. For example, Ramírez-Sevilla et al. reported side effects in 1.36% of 1104 patients, consisting of dry mouth, gastritis, and mild nausea. No adverse events were reported in patients treated with autovaccines [10]. However, it should be noted that the randomized controlled trial reported a higher rate of adverse effects—40.8% in the immunoprophylaxis group and 50% in the control group. However, the percentages of patients who withdrew from the study due to adverse effects were 2.5% and 6.2% in the placebo and MV240 groups, respectively. The study design permitted systematic assessment of all symptoms and signs during follow-up. Nevertheless, the authors stated that most adverse events were not treatment-related, as reflected in the high prevalence of side effects in the placebo group [16,24]. The study by Bauer et al., which compared immunotherapy using OM-89 with placebo, reported an even higher incidence of adverse events, with 13% considered treatment-related [29]. The Cochrane review by Albert et al. reported that up to 20% of patients required withdrawal, and some studies reported a prevalence of 28% adverse effects in the placebo group and up to 10% severe adverse effects [34]. Therefore, future studies are needed to more accurately determine the adverse effects directly attributable to sublingual immunoprophylaxis.

The strength of this review lies in its focus on one type of immunoprophylaxis—sublingual whole-bacteria formulations—and in its analysis of both MV140 and autovaccine formulations with independently reported outcomes. The main limitation is the risk of bias in the included studies, as only one was a randomized controlled trial; most studies compared UTI incidence before and after treatment. Observed improvements may be influenced by changes in risk factors or concomitant measures. Furthermore, in observational comparative studies with low-dose antibiotics, treatment allocation often depended on physician recommendation, potentially limiting comparability between groups. Moderate heterogeneity was observed among studies. A longer follow-up is recommended, as UTIs frequently recur during this period. Adverse events should be reported systematically, as reported rates varied considerably, with the highest percentage observed in the RCT. Robust randomized controlled trials are necessary to provide firm recommendations regarding immunoprophylaxis with oral formulations of inactivated whole bacteria for rUTI prevention. Following this route, a multicenter double-blind randomized controlled trial evaluating MV140 was recently published as a protocol [25].

## 4. Materials and Methods

### 4.1. Definitions Used

A comprehensive literature review was conducted. PubMed, Cochrane CENTRAL, and Embase libraries were searched with the terms ‘‘recurrent urinary tract infections’’ and ‘‘Uromune’’ or ‘‘MV140’’ or ‘‘sublingual vaccine’’ or “whole cell” or “bacterial vaccine”. References of the included studies and previous reviews were also examined to identify additional relevant studies.

### 4.2. Study Variables and Outcomes

The primary aim of this study was to evaluate the effectiveness of sublingual immunoprophylaxis with inactivated whole bacteria (MV140 formula and autovaccine) for the prevention of rUTIs. Effectiveness was assessed using the following criteria:

- The number of UTIs experienced during follow-up.
- The proportion of patients remaining UTI-free during follow-up.
- Comparisons were made against no treatment or prophylaxis with low-dose antibiotics.

Secondary outcomes included the following for patients receiving sublingual immunoprophylaxis, placebo, or low-dose antibiotic prophylaxis:

- The proportion of patients experiencing fewer than three UTIs during follow-up;
- Infection-free interval;
- Microbiological patterns, including rates of multidrug-resistant organisms (MDROs);
- Quality of life;
- Adverse events;
- Comparative efficacy between MV140 and autovaccine.

The systematic review was preregistered at PROSPERO. The detailed review protocol can be viewed under CRD number 420251003894 at https://www.crd.york.ac.uk/PROSPERO/view/CRD420251003894 (accessed on 4 May 2025).

### 4.3. Literature Search

A systematic review was conducted according to the Preferred Reporting Items for Systematic Reviews and Meta-Analyses (PRISMA) guidelines (Supplementary Materials) [36,37]. The following PICO question was formulated: Patients (adult patients with recurrent urinary tract infections), Intervention (treatment using sublingual immunoprophylaxis with whole bacteria inactivated via heat), Control (prophylaxis using a low dose of antibiotics, placebo, or number of infections before treatment using sublingual immunoprophylaxis with whole bacteria inactivated via heat), Outcome (rates of urinary tract infections, time free of infections, microbiological patterns, quality of life assessment, and side effects).

Search Strategy: Eligible studies were English-language manuscripts that addressed the prevention and management of rUTIs using sublingual dead whole bacteria treatment. The inclusion criteria were adult patients (older than 16 years old), including males and females, and studies evaluating the effect of sublingual immunoprophylaxis for rUTI prevention.

### 4.4. Eligibility of Studies

Eligible study designs included randomized controlled trials (RCTs), non-randomized controlled trials (non-RCTs), observational studies (prospective or retrospective), cross-sectional studies, case–control studies, and single-arm studies with ≥25 patients. When multiple publications evaluated the same cohort, the larger or more comprehensive study was included. Excluded studies comprised case reports, expert opinions, commentaries, editorials, and conference abstracts. Only studies documenting the effectiveness and safety of sublingual inactivated whole-bacteria immunoprophylaxis for rUTI prevention were included.

### 4.5. Selection of Studies and Data Extraction

Data extraction (selection and coding)

Study selection: Studies were selected in a two-step process. Titles and abstracts were first screened against the predefined inclusion criteria. Second, the full texts of potentially eligible studies were assessed for eligibility using the same criteria. Two authors independently screened and selected the studies.

Data extraction: Data extraction was undertaken by one author. The extracted data were checked by a second author. Any discrepancies were resolved through discussion.

Extracted data included the following:

- Study characteristics (design, objective, sample size, follow-up duration, publication year, and statistical methods).
- Type of immunoprophylaxis with sublingual dead whole bacteria: MV140 formula or autovaccine.
- Definition of rUTIs.
- Definition of UTI episode.
- Treatment indicated (duration and dose).
- Incidence of UTIs.
- No UTIs during follow-up.
- Prevalence of more than three UTIs during follow-up.
- Time free of UTIs.
- Microorganism patterns, including the rate of MDRO.
- Quality of life evaluation.
- Adverse effects.

### 4.6. Risk of Bias Assessment

Risk of bias was assessed using the Cochrane Risk of Bias tool for RCTs, considering the following domains: selection bias, performance bias, classification of interventions, missing data, outcome measurement, and reporting bias. For non-randomized studies, the ROBINS-I tool (Risk Of Bias in Non-randomized Studies of Interventions) was employed.

### 4.7. Meta-Analysis

A meta-analysis was performed using a random-effects model. Between-study heterogeneity was assessed visually using forest plots and quantified using the I^2^ statistic (95% CI) and the Chi-square test for heterogeneity. The analysis included the estimated average log odds ratios based on the random-effects model and the Q-test for heterogeneity. All analyses were conducted using SPSS Statistics for Mac, version 29.0.2.0 (IBM Corporation).

## 5. Conclusions

Although the quality of evidence regarding the efficacy of whole inactivated bacteria immunoprophylaxis for the prevention of rUTIs is limited, current data indicate that it may confer a beneficial effect. This systematic review suggests that MV140 and autovaccine formulations, administered sublingually as whole inactivated bacteria, represent safe and effective non-antibiotic alternatives for reducing UTI frequency and prolonging UTI-free intervals in women with recurrent urinary tract infections. Nonetheless, well-designed prospective randomized controlled trials are required to confirm these findings.

## Figures and Tables

**Table 1:** Characteristics of comparative studies.

Study	Type of Study	ITUr Definition	ITU Diagnosis	Men	Women	Menopausal	Number Patients	Patients Treatment	Number Controls	Controls Treatment
Lorenzo-Gómez et al. 2022 [16]	Randomized Controlled Trial	2 UTIs 6 months or 3 UTIs 12 months	Symptoms and urine culture >10^3^ cfu/mL	0 (0%)	215 (100%)	145 (67.4%)	139	MV140; 3 or 6 months	76	Placebo 6 months
Lorenzo-Gómez et al. 2013 [11]	Observational Prospective Comparative	2 UTIs 6 months or 3 UTIs 12 months	Symptoms and positive urine culture	0 (0%)	159 (100%)	64 (40.3%)	159	MV140; 3 months	160	SMX200-TMP40 6 months
Lorenzo-Gómez et al. 2015 [12]	Retrospective Cohort Study	2 UTIs 6 months or 3 UTIs 12 months	Symptoms and urine culture >10^3^ cfu/mL	0 (0%)	360 (100%)	170 (47.2%)	360	MV140; 3 months	339	SMX200-TMP40 or Furantoin100 6 months
2023 Ramírez-Sevilla et al. [18]	Prospective Observational Comparative	2 UTIs 6 months or 3 UTIs 12 months	Symptoms and positive urine culture	53 (14.1%)	324 (85.9%)	282 (87%)	251 (MV140 125, Autovaccine 126)	MV140/Autovaccine; 3 months	126	Antibiotics 6 months (cefuroxime, fosfomycin, nitrofurantoin)

UTIs: Urinary tract infections. rUTIs: Recurrent urinary tract infections. cfu: colony-forming units; The percentage of menopausal Women is calculated from the total of women included. SMX200-TMP40: sulfamethoxazole (SMX) 200 mg and trimethoprim (TMP) 40 mg. MV140 formula consist of a suspension of whole-cell, heat-inactivated bacteria with equal amounts of *Escherichia coli*, *Klebsiella pneumoniae*, *Proteus vulgaris* and *Enterococcus faecalis*. Autovaccine consist of heat-inactivated immunoprophylaxis prepared according to the results of the urine culture.

**Table 2:** Findings in the comparative studies.

	Lorenzo-Gómez et al. 2022 [16]	Lorenzo-Gómez et al. 2013 [11]	Lorenzo-Gómez et al. 2015 [12]	Ramírez-Sevilla et al. 2023 [18]
Baseline UTIs Cases [mean (SD)]	6 (1)	3.2 (0.5)	6 (1)	
Baseline UTIs Controls [mean (SD)]	6 (1)	3.1 (0.2)		
3 months UTIs Controls [mean (SD)]	3 (2.7)	1.6 (0.1)		
3 months No UTIs Cases [n (%)]		101 (63.5%)	290 (81%)	99 (39.4%)
3 months No UTIs Controls [n (%)]		9 (5.6%)	9 (3%)	40 (31.7%)
3 months > 3 UTIs Cases [n (%)]				7 (2.8%)
3 months > 3 UTIs Controls [n (%)]				7
6 months UTIs reduction (%) MV140		86%		
6 months UTIs Cases [mean (SD)]		0.72 (0.2)		
6 months UTIs Controls [mean (SD)]		3.71 (0.3)		
6 months No UTIs Cases [n (%)]	93 (66%)		290 (81%)	41 (16.3%)
6 months No UTIs Controls [n (%)]	25 (32.9%)		9 (3%)	15 (11.9%)
6 months > 3 UTIs Cases [n (%)]				25 (10%)
6 months > 3 UTIs Controls [n (%)]				22 (17.5%)
12 months UTIs reduction (%) MV140		77%		
12 months UTIs Cases [mean (SD)]	0.0 (2)	1.35 (0.2)		
12 months UTIs Controls [mean (SD)]	6 (2)	5.75 (0.4)		
12 months No UTIs Cases [n (%)]	79 (56.8%)	90 (56.6%)	325 (90.3%)	
12 months No UTIs Controls [n (%)]	19 (25%)	4 (2.5%)	0 (0%)	
12 months > 3 UTIs Cases [n (%)]	19 (13.7%)			
12 months > 3 UTIs Controls [n (%)]	42 (55.2%)			
15 months No UTIs Cases [n (%)]	57 (41%)	55 (34.6%)		
15 months No UTIs Controls [n (%)]	16 (21.1%)	0 (0%)		
Median Time to UTIs Cases (days)	275		180	
Median Time to UTIs Controls (days)	48		19	
Resistant Microorganisms Cases (%)			31%	
Resistant Microorganisms Controls (%)			45%	
Side Effects Cases [n (%)]	62 (40.8%)	0 (0%)	0 (0%)	0 (0%)
Side Effects Controls [n (%)]	39 (50.0%)	NR	NR	3 (2.4%)

UTIs: Urinary tract infections. [mean (SD)]: Mean and Standard Deviation. [n (%)]: Number and percentage. NR: No data reported.

**Table 3:** Characteristics of observational studies.

Study	Type of Study	ITUr Definition	ITU Diagnosis	Men	Women	Menopausal	Number Patients	Patients Treatment
Yang et al. 2018 [13]	Observational Prospective	3 UTIs 12 months	Symptoms and positive urine culture	0 (0%)	75 (100%)	NR	75	MV140; 3 months
Ramírez-Sevilla et al. 2024 [10]	Observational Prospective	2 UTIs 6 months or 3 UTIs 12 months	Symptoms and urine culture > 10^5^ cfu/mL	0 (0%)	1104 (100%)	730 (66.1%)	1104	MV140 (648) or Autovaccine (456); 3 months
Lorenzo-Gómez et al. 2021 [14]	Observational Prospective	2 UTIs 6 months or 3 UTIs 12 months	Symptoms and positive urine culture	40 (20%)	160 (80%)	160 (100%)	200	MV140 (140) or Autovaccines (60); 3–6 months
Bonillo-García et al. 2025 [23]	Observational Prospective	2 UTIs 6 months or 3 UTIs 12 months	Symptoms and urine culture > 10^2^ cfu/mL in patients with intermittent catheterization and >10^4^ cfu/mL in clean voided specimens	48 (68.6%)	22 (31.4%)	NR	70	Autovaccine; 3 months
Sánchez-Ramón et al. 2021 [15]	Observational Prospective	3 UTIs 12 months	Symptoms and positive urine culture	0 (%)	27 (100%)	NR	27	MV140; 3 months
Carrión-López et al. 2022 [17]	Observational Prospective	3 UTIs 12 months	Symptoms and positive urine culture	0 (0%)	166 (100%)	125 (75.4%)	166	MV140; 3 months
Lopes et al. 2024 [19]	Observational Prospective	3 UTIs 12 months	Symptoms and urine culture > 10^5^ cfu/mL	24 (22.2%)	84 (77.8%)	42 (38.9%)	108	MV140; 3 months
Nickel et al. 2024 [20]	Observational Prospective	3 UTIs 12 months	Symptoms and urine culture > 10^5^ cfu/mL	0 (0%)	64 (100%)	NR	64	MV140; 3 months
Iftimie et al. 2025 [22]	Observational Retrospective	2 UTIs 6 months or 3 UTIs 12 months	Symptoms and urine culture > 10^5^ cfu/mL	20 (40.8%)	29 (59.2%)	20 (69%)	49	Autovaccine; 3 months
Hernández-Sánchez et al. 2025 [21]	Prospective Observational	2 UTIs 6 months or 3 UTIs 12 m	Symptoms and positive urine culture	NR	NR	NR	732	Autovaccine; 3 months

UTIs: Urinary tract infections. rUTIs: Recurrent urinary tract infections. cfu: colony-forming units. NR: No data reported. The percentage of menopausal Women is calculated from the total of women included. MV140 formula consist of a suspension of whole-cell, heat-inactivated bacteria with equal amounts of *Escherichia coli*, *Klebsiella pneumoniae*, *Proteus vulgaris* and *Enterococcus faecalis*. Autovaccine consist of heat-inactivated immunoprophylaxis prepared according to the results of the urine culture.

**Table 4:** Findings in the observational studies.

	Yang et al. 2018 [13]	Ramírez-Sevilla et al. 2024 [10]	Lorenzo-Gómez et al. 2021 [14]	Bonillo-García et al. 2025 [23]	Sánchez-Ramón et al. 2021 [15]	Carrión-López et al. 2022 [17]	Lopes et al. 2024 [19]	Nickel et al. 2024 [20]	Iftimie et al. 2025 [22]	Hernández-Sánchez et al. 2025 [21]
Baseline UTIs Cases [mean (SD)]			5 (2)		5 (2)	6.19 (2.1)	4.79 (1.39)	6.8	3.73 (0.97)	
3 months UTIs reduction (%)		71.70%								
3 months UTIs Cases [mean (SD)]						2.81 (2.5)				2
3 months No UTIs Cases [n (%)]	63 (85%)	458 (41.5%)		48 (69.12%)		124 (74.4%)		31 (48.4%)	44 (89.8%)	
3 months > 3 UTIs Cases [n (%)]		92 (8.3%)								
6 months UTIs reduction (%)		64.70%								
6 months No UTIs Cases [n (%)]	60 (80%)	287 (26%)		30 (42.65%)		113 (68.1%)				
6 months > 3 UTIs Cases [n (%)]		195 (17.7%)								
12 months UTIs reduction (%)	59 (78%)	108 (9.8%)	84 (42%)	15 (20.59%)		87 (52%)	41 (38%)	26 (40.6%)	24 (49%)	
12 months UTIs Cases [mean (SD)]			0.2 (0.2)		1 (1)		1.58 (0.33)		0.98 (1.36)	
12 months No UTIs Cases [n (%)]										
12 months > 3 UTIs Cases [n (%)]		636 (57.6%)	82 (41%)				36 (34%)		7 (14.3%)	
24 months No UTIs Cases [n (%)]						74 (44.5%)				
Case Median Time to UTIs (days)	60			318.6						
Median Antibiotics Consumed					0.5	2.89				
Side Effects Cases [n (%)]	7 (9.3%)	15 (1.36%)	0 (0%)		0 (0%)	2 (1.2%)	5 (4.6%)	9 (14%)	0 (0%)	

UTIs: Urinary tract infections. [mean (SD)]: Mean and Standard Deviation. [n (%)]: Number and percentage. NR: No data reported.

**Table 5:** Characteristics of studies using autovaccine.

Study	Type of Study	ITUr Definition	Men	Women	Menopausal	Number Patients	Patients MV140 Formula	Patients Autovaccine	Controls
Ramírez-Sevilla et al. 2023 [18]	Observational Prospective Comparative	2 UTIs 6 months or 3 UTIs 12 months	53 (14.1%)	324 (85.9%)	282 (87%)	251	125	126	126 Antibiotic 6 months
Ramírez-Sevilla et al. 2024 [10]	Observational Prospective	2 UTIs 6 months or 3 UTIs 12 months	0 (0%)	1104 (100%)	730 (66.1%)	1104	648	456	
Lorenzo-Gómez et al. 2021 [14]	Observational Prospective	2 UTIs 6 months or 3 UTIs 12 months	40 (20%)	160 (80%)	160 (100%)	200	140	60	
Bonillo-García et al. 2025 [23]	Observational Prospective	2 UTIs 6 months or 3 UTIs 12 months	48 (68.6%)	22 (31.4%)	NR	70	0	70	
Iftimie et al. 2025 [22]	Observational Retrospective	2 UTIs 6 months or 3 UTIs 12 months	20 (40.8%)	29 (59.2%)	20 (40.8%)	49	0	49	
Hernández-Sánchez et al. 2025 [21]	Observational Prospective	2 UTIs 6 months or 3 UTIs 12 months	NR	NR	NR	732	0	732	444 Antibiotic

UTIs: Urinary tract infections. rUTIs: Recurrent urinary tract infections. NR: No data reported. The percentage of menopausal Women is calculated from the total of women included. MV140 formula consist of a suspension of whole-cell, heat-inactivated bacteria with equal amounts of *Escherichia coli*, *Klebsiella pneumoniae*, *Proteus vulgaris* and *Enterococcus faecalis*. Autovaccine consist of heat-inactivated immunoprophylaxis prepared according to the results of the urine culture.

**Table 6:** Findings of the studies using autovaccine.

	Ramírez-Sevilla et al. 2023 [18]	Ramírez-Sevilla et al. 2024 [10]	Lorenzo-Gómez et al. 2021 [14]	Bonillo-García et al. 2025 [23]	Iftimie et al. 2025 [22]	Hernández-Sánchez et al. 2025 [21]
Baseline UTIs Cases [mean (SD)]			5 (2)		3.73 (0.97)	
3 months UTIs Autovaccine [mean]						2
3 months No UTIs Cases [n (%)]	99 (39.4%)	458 (41.5%)		48 (69.12%)	44 (89.8%)	
3 months No UTIs MV140 [n (%)]	61 (48.4%)	302 (46.6%)				
3 months No UTIs Autovaccine [n (%)]	38 (30.4%)	156 (34.2%)		48 (69.12%)	44 (89.8%)	
3 months No UTIs Control [n (%)]	40 (31.7%)					
3 months > 3 UTIs Cases [n (%)]	7 (2.8%)	92 (8.3%)				
3 months > 3 UTIs MV140 [n (%)]	3 (2.4%)	27 (4.2%)				
3 months > 3 UTIs Autovaccine [n (%)]	4 (3.2%)	65 (14.3%)				
3 months > 3 UTIs Control [n (%)]	7 (5.6%)					
6 months No UTIs Cases [n (%)]	41 (16.3%)	287 (26%)		30 (42.65%)		
6 months No UTIs MV140 [n (%)]	29 (23%)	193 (29.8%)				
6 months No UTIs Autovaccine [n (%)]	12 (9.6%)	94 (20.6%)		30 (45.65%)		
6 months No UTIs Control [n (%)]	15 (11.9%)					
6 months > 3 UTIs Cases [n (%)]	25 (10%)	195 (17.7%)				
6 months > 3 UTIs MV140 [n (%)]	10 (7.9%)	75 (11.6%)				
6 months > 3 UTIs Autovaccine [n (%)]	15 (12%)	120 (26.4%)				
6 months > 3 UTIs Control [n (%)]	22 (17.5%)					
12 months UTIs Reduction Autovaccine (%)					73.72%	
12 months UTIs MV140 [mean (SD)]			0.1 (0.3)			
12 months UTIs Autovaccine [mean (SD)]			0.3 (0.2)		0.98 (1.36)	
12 months No UTIs Cases [n (%)]		108 (9.8%)	84 (42%)	15 (20.59%)	24 (59%)	
12 months No UTIs MV140 [n (%)]		63 (9.7%)	84 (60%)			
12 months No UTIs Autovaccine [n (%)]		46 (10%)	0 (0%)	15 (20.59%)	24 (49%)	
12 months > 3 UTIs Cases [n (%)]		636 (57.6%)	82 (41%)		7 (14.3%)	
12 months > 3 UTIs MV140 [n (%)]		364 (56.1%)	28 (20%)			
12 months > 3 UTIs Autovaccine [n (%)]		275 (60.2%)	54 (90%)		7 (14.3%)	
Median Time to UTIs Cases (days)				318.6		
Side Effects Cases [n (%)]	0 (0%)	15 (1.36%)	0 (0%)		0 (0%)	

UTIs: Urinary tract infections. [mean (SD)]: Mean and Standard Deviation. [n (%)]: Number and percentage. NR: No data reported.

## Data Availability

No new data were created or analyzed in this study. Data sharing is not applicable to this article.

## Supplementary Materials

The following supporting information can be downloaded at: https://www.mdpi.com/article/10.3390/antibiotics15010006/s1, PRISMA 2020 Checklist [36].

## Abbreviations

The following abbreviations are used in this manuscript:

UTIs	Urinary tract infections
rUTIs	Recurrent urinary tract infections
RCT	Randomized controlled trials
PRISMA	Preferred Reporting Items for Systematic reviews and Meta-Analyses
cfu	Colony-forming units
mL	Milliliter
SMX200	Sulfamethoxazole
TMP40	Trimethoprim
Mean (SD)	Mean and Standard Deviation
n(%)	Number and percentage
NR	No data reported
CI	confidence interval
ROBINS-I	Risk Of Bias in Non-randomised Studies of Interventions
SPSS	Statistical Package for the Social Sciences
